# N6-Methyladenosine RNA Modification in Inflammation: Roles, Mechanisms, and Applications

**DOI:** 10.3389/fcell.2021.670711

**Published:** 2021-06-04

**Authors:** Jiahui Luo, Tao Xu, Kai Sun

**Affiliations:** ^1^The Center for Biomedical Research, Tongji Hospital, Tongji Medical College, Huazhong University of Science and Technology, Wuhan, China; ^2^Department of Rehabilitation, Tongji Hospital, Tongji Medical College, Huazhong University of Science and Technology, Wuhan, China; ^3^Department of Orthopedics, Tongji Hospital, Tongji Medical College, Huazhong University of Science and Technology, Wuhan, China

**Keywords:** N6-methyladenosine, RNA modification, epigenetics, inflammation, inflammatory disease

## Abstract

N6-methyladenosine (m6A) is the most prevalent internal mRNA modification. m6A can be installed by the methyltransferase complex and removed by demethylases, which are involved in regulating post-transcriptional expression of target genes. RNA methylation is linked to various inflammatory states, including autoimmunity, infection, metabolic disease, cancer, neurodegenerative diseases, heart diseases, and bone diseases. However, systematic knowledge of the relationship between m6A modification and inflammation in human diseases remains unclear. In this review, we will discuss the association between m6A modification and inflammatory response in diseases, especially the role, mechanisms, and potential clinical application of m6A as a biomarker and therapeutic target for inflammatory diseases.

## Introduction

In recent years, epitranscriptomic modifications have been recognized as essential regulators of various physiological processes and disease progression. RNA modification is an example of dynamic epigenetic regulation. Early RNA modification studies have revealed important functions of RNA modification in translation and splicing. These studies focused on abundant non-coding RNAs, such as rRNA, tRNA, and small nuclear RNA (Jung and Goldman, 2018). The tRNA modification N1-methyladenosine (m1A) regulates the associations between tRNA and polysomes to stabilize tRNA tertiary structure and modify translation (Li et al., 2016). Pseudouridine (Ψ) in snRNA participates in mRNA splicing, whereas Ψ in rRNA ensures translational fidelity (Xiong et al., 2017).

N6-methyladenosine (m6A) is the most prevalent internal mRNA modification, which was first discovered in the early 1970s (Dubin and Taylor, 1975). m6A participates in almost all processes involving mRNA metabolism, including RNA transcription, translation, and degradation (Roundtree et al., 2017a; Nachtergaele and He, 2018). Similar to DNA methylation, RNA m6A modification is a dynamic and reversible process (Zhao et al., 2020). m6A is installed by the methyltransferase complex (MTC) and removed by demethylases, which regulate the post-transcriptional expression of target genes (Batista, 2017; Dai et al., 2018). In molecular processing, m6A is involved in many steps of RNA metabolism, including pre-mRNA splicing, mRNA translation, nuclear export, mRNA degradation, and non-coding RNA biogenesis (Liu et al., 2017; Shi et al., 2017; Liu and Gregory, 2019).

Inflammation is a complex physiological reaction to microorganisms, autoimmunity, allergies, metabolism, and physical damage, all of which produce different types of inflammatory responses (Hawiger and Zienkiewicz, 2019). In acute inflammation, the initial response of the body to a stimulus is achieved by increasing white blood cell migration and plasma leakage from the blood to the site of injury (Bayarsaihan, 2011; Varela et al., 2018). Chronic inflammation progresses slowly, occurs for a longer duration, and can cause many diseases, including periodontal disease and diabetes (Dunning, 2009). Inflammatory response requires a complex regulatory network to achieve signal- and gene-specific levels of function (Medzhitov and Horng, 2009) for activating specific genes for antimicrobial defense, immune response, and tissue repair and remodeling (Medzhitov, 2008). Emerging evidence suggests that epigenetic modifications are involved in inflammatory response. Macrophages play critical roles in various inflammatory diseases, including obesity and arthritis. Chromatin modifications have been reported to participate in the regulation of macrophage phenotype (Nemat et al., 2009). In addition, DNA methylation and covalent histone modifications of transcription factors, including NF-κB and the STAT families, have been found to modulate inflammatory genes (Medzhitov and Horng, 2009). However, the role of RNA modification in regulating inflammation and anti-inflammatory gene expression has only been recently elucidated. In this review, we will discuss the association between m6A methylation and inflammatory response in diseases, especially its role, mechanism, and potential clinical application in inflammation-related diseases.

### RNA m6A Methylation

**FIGURE 1:**
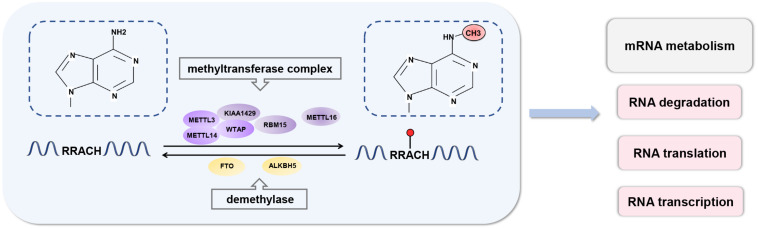
Dynamic process of RNA N6-methyladenosine methylation. RNA m6A modification is a dynamic and reversible process. m6A can be installed by the methyltransferase complex and removed by demethylases. m6A modifications are enriched in the 3′-untranslated regions (UTRs) near the stop codons of mRNA and within internal long exons, mainly with a consensus sequence of RRACH. m6A participates in almost all processes in mRNA metabolism, including RNA transcription, translation, and degradation.

In mammals, approximately 0.1–0.4% of adenosine in mRNA is modified by m6A, and each transcript has an average of 2–3 m6A modification sites (Desrosiers et al., 1974; Wei et al., 1975; Fu et al., 2014). m6A modifications are enriched in the 3′-untranslated regions (UTRs) near the stop codons of mRNA and within internal long exons, mainly with a consensus sequence of RRACH (R = G or A; H = A, C, or U) (Kane and Beemon, 1985; Dominissini et al., 2012). There are three types of regulators involved in m6A modifications and are referred to as “writers,” “erasers,” and “readers” (Figure 1).

“Writers” traditionally refer to a highly conserved mRNA methyltransferase complex that consists of methyltransferase-like 3 (METTL3), methyltransferase-like 14 (METTL14), and Wilms tumor suppressor-1-associated protein (WTAP) (Ping et al., 2014; Schwartz et al., 2014). Both METTL3 and METTL14 contain a S-adenosylmethionine-binding motif (Wang et al., 2016). METTL3 acts as a major catalytic component that modulates m6A modification (Barbieri et al., 2017). METTL14 is a pseudomethyltransferase that serves as an accessory component of METTL3 and participates in substrate recognition (Weng et al., 2018). They form a stable heterodimer and co-localize in nuclear speckles in a 1:1 ratio. WTAP is the main regulatory component and interacts directly with METTL3. It promotes methylation and ensures the nuclear location of the core writer complex (Liu et al., 2014; Schöller et al., 2018). The other known writers are methyltransferase-like 16 (METTL16) (Pendleton et al., 2017; Warda et al., 2017), KIAA1429 (Schwartz et al., 2014), and RBM15 (Moindrot et al., 2015). METTL16 methylates long non-coding RNA and U6 small nuclear RNA and regulates S-adenosylmethionine homeostasis (Pendleton et al., 2017; Warda et al., 2017). KIAA1429 and RBM15 are also components of methyltransferase complexes. KIAA1429 is required for full methylation in mammals (Schwartz et al., 2014). RBM15 mediates the formation of m6A in X-inactive specific transcripts and cellular mRNAs (Moindrot et al., 2015). However, the detailed functions of these components are still poorly understood, and there are likely other mechanisms that remain to be investigated.

Reversible modifications of m6A can be removed by “erasers,” such as fat mass and obesity-related protein (FTO) and ALKB homolog 5 protein (ALKBH5) (Jia et al., 2011; Zheng et al., 2013). These are ferrous iron- and α-ketoglutarate-dependent demethylases, which can oxidatively remove m6A methylated groups from mRNA (Chen and Wong, 2020). Both FTO and ALKBH5 belong to the ALKB family of dioxygenases (Fedeles et al., 2015). FTO sequentially oxidizes m6A to N6-hydroxymethyladeosine and N6-formyladenosine, which can be further hydrolyzed to adenine (Wei et al., 2018). ALKBH5 directly catalyzes the removal of m6A modifications. Recently, another m6A demethylase, ALKBH3, that preferentially act on m6A in tRNA rather than mRNA or rRNA has been reported (Ueda et al., 2017). Given that m6A is a dynamic modification in response to stimuli, demethylases ensure the equilibrium of m6A modifications in the transcriptome.

**FIGURE 2:**
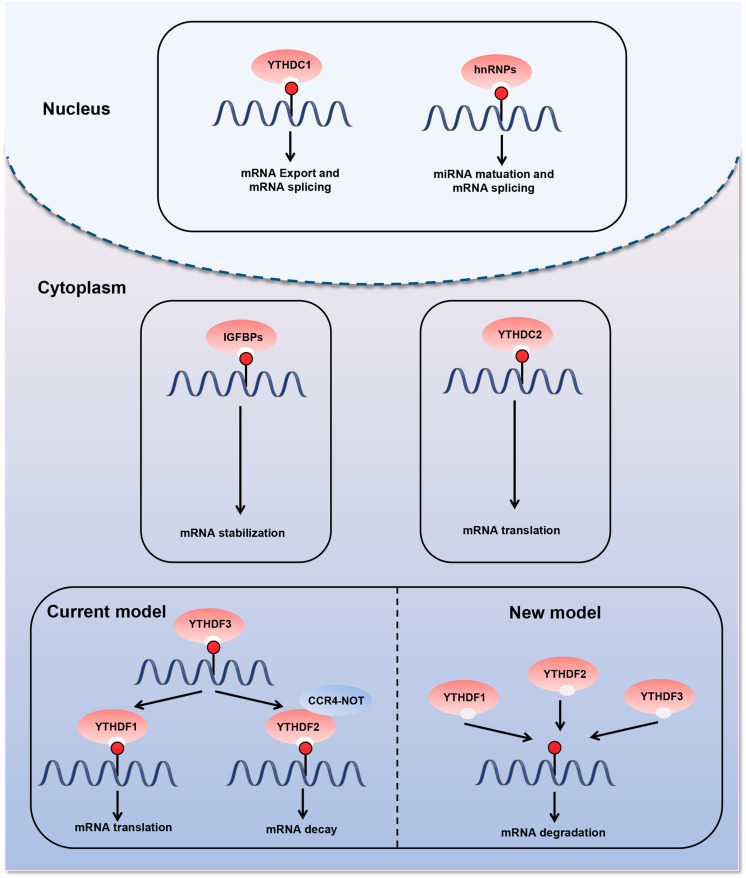
Diverse biological consequences of m6A methylation by different m6A “readers.” m6A “readers” can recognize and bind to the m6A modification sites in RNA. They play an important role in different biological functions. The first m6A “readers” identified were YTHDF1, YTHDF2, YTHDF3, YTHDC1, and YTHDC2, which contain a conserved YTH domain (YT521-B homology). Moreover, three heterogeneous nuclear ribonucleoproteins (hnRNPs) are common “readers,” namely hnRNPC, hnRNPG, and hnRNPA2B1. m6A “readers” are involved in various steps of RNA metabolism, including pre-mRNA splicing, mRNA translation, nuclear export, and mRNA degradation.

The last type of m6A regulatory proteins are “readers,” which can recognize and bind to the m6A modification site in RNA and play an important role in different biological functions (Figure 2). The first m6A “reader” proteins identified were YTHDF1, YTHDF2, YTHDF3, YTHDC1, and YTHDC2, which contain a conserved YTH domain (YT521-B homology) (Liao et al., 2018). The YTH domain functions as a module that recognizes modifications and directly binds to m6A-modified RNA in the RRm6ACH consensus sequence (Roundtree et al., 2017a). YTHDF2, the first “reader” identified, accelerates mRNA degradation by binding m6A at the 3′ UTR and localizing the targeted mRNA to processing bodies (Ries et al., 2019). In addition, YTHDF2 can induce mRNA degradation through the recruitment of the CCR4-NOT deadenylation machinery (Du et al., 2016). Moreover, YTHDF2 has been found to prevent m6A modification in the 5′ UTR of the FTO protein by binding to the m6A site in the nucleus, thus promoting RNA translation in a cap-independent manner (Zhou et al., 2015). YTHDF1 binds to m6A sites around the stop codon and enhances mRNA translation by recruiting the eIF3 translation initiation complex (Wang et al., 2015; Chen and Wong, 2020). YTHDF3 has a fine-tuning effect on the RNA accessibility of YTHDF1 and YTHDF2 (Hu B. B et al., 2019). YTHDF3 can interact with YTHDF1 to improve RNA translation efficiency and combine with YTHDF2 to promote RNA degradation (Li et al., 2017; Shi et al., 2017). Recent—studies have revealed a new model describing the functions of YTHDF proteins. YTHDF proteins function together to mediate mRNA degradation, and they show identical binding to all m6A sites in mRNA (Zaccara and Jaffrey, 2020). Each YTHDF paralog can compensate for the function of other YTHDF paralogs (Zaccara and Jaffrey, 2020). Another study confirmed the context-dependent functional compensation between YTHDF proteins (Lasman et al., 2020). YTHDC1 regulates RNA splicing and controls the nuclear export of its target RNA (Meyer and Jaffrey, 2017; Roundtree et al., 2017b). YTHDC2 promotes translation elongation by interacting with RNA helicases (Hsu et al., 2017). Three heterogeneous nuclear ribonucleoproteins (hnRNPs) are common “readers,” namely hnRNPC, hnRNPG, and hnRNPA2B1 (Alarcón Claudio et al., 2015; Liu et al., 2015; Meyer Kate et al., 2015). These proteins indirectly bind to transcripts through the “m6A switch” mechanism. This mechanism relies on the ability of m6A to thermodynamically destabilize into short double helices. In this way, the single-stranded hnRNP binding motif is exposed and provides access to RNA-binding proteins. hnRNPC and hnRNPG influence mRNA localization and alternative splicing (König et al., 2010; Liu et al., 2015, 2017). HnRNPA2B1 promotes the transcription of precursor miRNA by binding to m6A-containing primary miRNAs and interacting with the microRNA microprocessor complex (Alarcón Claudio et al., 2015). Recently, the IGFBP family IGFBP1-3 has been identified as a distinct family of m6A “readers” (Li et al., 2019; Müller et al., 2019; Wang S. et al., 2019). The binding motif of these proteins (UGGAC) overlaps with the consensus sequence of m6A. In addition, these proteins contain common RNA-binding domains that recognize m6A-containing transcripts. IGFBPs recruit RNA stabilizers that protect m6A-containing mRNA from degradation, thereby promoting the expression of target transcripts (He et al., 2019).

### Physiological Role of m6A RNA Modification

Under normal conditions, m6A modification interferes with RNA recognition (Karikó et al., 2005). Viruses can use m6A modification to escape host immune recognition because of the presence of widely distributed m6A modifications in viral mRNA (Tang et al., 2021). In addition, mounting evidence has demonstrated m6A modifications in viral RNA (Durbin et al., 2016; Lu et al., 2020), host circRNAs (Chen et al., 2019), and normal endogenous ssRNA (Gao et al., 2020). m6A can serve as a key factor for the innate immune system to avoid abnormal immune recognition.

Innate immunity is activated immediately after the RNA recognition process, and it induces the production of cytokines, such as type I interferons (IFNs) (Winkler et al., 2019). m6A modification regulates the innate immune response by targeting IFN. IFNB1 mRNA can be modified by m6A within both the coding sequence and the 3′ UTR (Rubio et al., 2018). IFN production triggered by dsDNA or human cytomegalovirus (HCMV) is mediated by m6A regulators. METTL14 depletion reduces virus reproduction and promotes IFNB1 mRNA accumulation, thus affecting pathways related to metabolic reprogramming, stress responses, and aging (Rubio et al., 2018). ALKBH5 depletion decreases IFNB1 mRNA production by regulating antiviral immune responses (Rubio et al., 2018). Moreover, it has been reported that IFNB mRNA is stabilized by repression of METTL3 or YTHDF2 after viral infection or inactivated virus stimulation (Winkler et al., 2019). Together, these findings reveal the important role of m6A as a negative regulator of IFN response, consequently facilitating viral propagation.

### Role of RNA m6A in Inflammation: From Cells to Disease Progression

**FIGURE 3:**
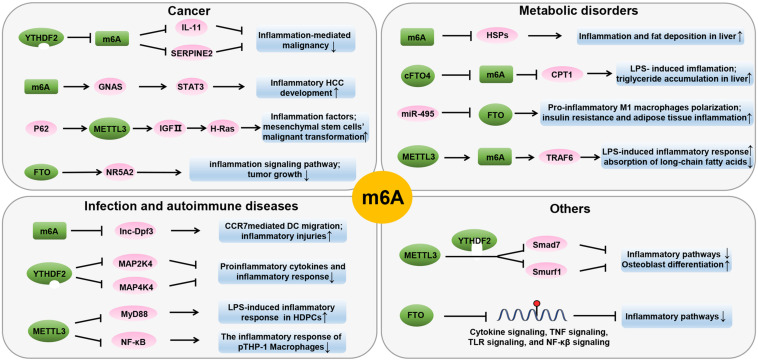
Role of m6A in inflammatory processes of various diseases states. m6A modification affects inflammation by regulating inflammation-related genes. RNA methylation is linked through numerous mechanisms and present in various inflammatory disease states, including autoimmunity, infection, metabolic diseases, and cancer.

m6A modifications can cause changes in inflammation-related genes during inflammation. A systematic study of m6A modified cross-linking, substrate genes, and modified regulation illustrated the mechanism of action of m6A in inflammation (Figure 3 and Table 1). In the following sections, we will elucidate the interaction between m6A modification and inflammation in the pathogenesis of various diseases, including metabolic disorders, autoimmune diseases, and malignant diseases.

**TABLE 1:** Roles of m6A in RNA metabolism and inflammatory processes.

Disease		Aberrant expression of m6A enzymes	Target RNA	Change of target RNA level	Effect of enzyme on target RNA	Role of m6A in diseases	Mechanism	References
Metabolic disorders	Obesity	FTO ↑	–			Increase fat mass, decrease energy expenditure	Promotes inflammatory profile in white adipose tissue; leads to higher CRP levels	Church et al., 2009; Zimmermann et al., 2011; Ligthart et al., 2016
	NASH	FTO ↑	–			Promotes fat accumulation, inflammation, and lipotoxicity in liver	Genetic silencing of FTO protects against palmitate-induced oxidative stress, mitochondrial dysfunction, ER stress, and apoptosis	Lim et al., 2016
Autoimmune diseases And Infection	RA	METTL3 ↑	(p)-NF-kB	↓	Phosphorylation and nucleus translocation	Increases inflammatory response in macrophages	Overexpression of METTL3 significantly attenuated the inflammatory response through the effect on NF-κB	Wang J. et al., 2019
	Dental pulp inflammation	METTL3 ↑	MyD88	↓	Splicing	Increases the expression of inflammatory cytokines	METTL3 regulate alternative splicing of MyD88; METTL3 depletion decreased the expression of inflammatory cytokines and the NF-kB signaling and MAPK signaling pathway	Feng et al., 2018
Cancer	HCC	YTHDF2 ↓	IL-11, SERPINE2	↑	Degradation	Promotes inflammation-mediated malignancy	YTHDF2 processed the decay of m6 A-containing IL11 and SERPINE2 mRNAs; YTHDF2 transcription succumbed to HIF-2α	Hou et al., 2019
	HCC	Unknown	GNAS	↑	Expression	Promotes LPS-induced HCC cell growth and invasion	Increasing m6A methylation of GNAS mRNA, GNAS promotes LPS-induced STAT3 activation in HCC cells through inhibiting long non-coding RNA TPTEP1 interacting with STAT3	Ding et al., 2020
	ICC	FTO ↓	NR5A2	↓	Expression	Regulates inflammatory gene expression and tumor growth	FTO knockdown inhibited the expression of NR5A2 which is associated with PDAC and transcriptionally regulates inflammatory gene expression	Rong et al., 2019
Others	Poststroke	FTO ↓	–			Modulates poststroke brain damage	Promotes cytokine signaling, TNF signaling, TLR signaling, and NF-kB signaling pathways	Chokkalla et al., 2019

### Metabolic Disorders

Under normal circumstances, the metabolic state of the human body is maintained under homeostasis. When normal metabolic processes are disrupted, metabolic disorders occur. m6A is involved in the regulation of gene expression and cellular metabolism. Accumulated data demonstrate the significant role of m6A methylation in metabolic homeostasis. Lipopolysaccharide (LPS) is considered an early trigger of inflammation and metabolic diseases. The *Fto* gene, which encodes m6A demethylase, has been found to be responsive to LPS and play a role in linking inflammation with metabolic responses (Zhang et al., 2016). LPS stimulation reduced the expression of carnitine palmitoyltransferase 1 (CPT1) in the liver, upregulated the truncated cFTO4 protein, and dramatically reduced m6A levels near the translation start site of CPT1 (Zhang et al., 2016). In addition, the core methyltransferase of m6A, METTL3, has been found to participate in the molecular mechanism of inflammation-induced malabsorption of long-chain fatty acids (LCFAs). METTL3 inhibits LPS-induced inflammatory response by exerting anti-malabsorption of LCFA activity *in vitro* (Zong et al., 2019). The depletion of *Mettl3* reduces the m6A level of *Traf6* mRNA, thereby decreasing the expression of TRAF6 and inhibiting the NF-κB and MAPK signaling pathways (Zong et al., 2019). These data suggest that m6A methylation plays an essential role in cellular metabolic homeostasis.

In obesity, extensive inflammation and stress reactions usually occur, which subsequently develop into chronic, low-grade, local inflammation and disruption of metabolic homeostasis (Ghemrawi et al., 2018). Studies have shown a correlation between inflammation and obesity. Experiments in obese mice have shown that the amounts of proinflammatory cytokines secreted by adipocytes and macrophages in adipose tissues are sufficient to disrupt insulin signaling (Olefsky and Glass, 2010). In addition, activation of the JNK and NF-κB signaling pathways causes obesity-induced inflammation and abnormal insulin action. The *Fto* gene is known to play an important role in the regulation of metabolic homeostasis, mainly owing to its association with body mass index (BMI). FTO overexpression increases food intake and promotes obesity (Church et al., 2010). miR-495 inhibited the expression of its target gene *Fto*, resulting in the promotion of the transformation of macrophages into M1-type pro-inflammatory macrophages, and aggravated insulin resistance and adipose tissue inflammation in type 2 diabetes mellitus (T2D) -mice (Hu et al., 2019a). In contrast, dominant point mutations in mouse *Fto* gene decreased fat mass, promoted energy expenditure, and improved the inflammatory profile of white adipose tissue of mutant mice (Church et al., 2009). Recent scientific literature has shed light on the genetic relationship between FTO and inflammation markers. Notably, studies have found that FTO is related to C-reactive protein (CRP) levels (Ligthart et al., 2016). Greater adiposity conferred by FTO SNPs leads to higher CRP levels (Welsh et al., 2010). Another study demonstrated that there was a significant positive correlation between hs-CRP, leptin, and broad BMI, but there was no significant difference in the FTO rs9939609 genotype (Zimmermann et al., 2011). Obesity is associated with a shorter telomere length (Zhou et al., 2017). Telomere length attrition may be affected by obesity-related inflammation, oxidative stress, and FTO gene pathways. The increase in BMI is genetically related to telomere length shortening, and low-grade inflammation may result from increased CRP levels (Rode et al., 2014). The association between the *Fto* gene and expression of inflammatory markers is unclear, and the underlying mechanisms remain to be elucidated.

Non-alcoholic fatty liver disease is accompanied by inflammation, which contributes to the development of fibrosis, cirrhosis, and hepatocellular carcinoma (HCC) (Sun et al., 2013). Chronic corticosterone exposure can induce liver inflammation and fibrosis and increase mRNA and m6A methylation of several heat shock proteins (HSPs) (Feng et al., 2020). HSPs are activated during acute stress response to exert a cytoprotective effect (Stolte et al., 2009), which has been confirmed to be related to m6A-mediated post-transcriptional regulation. In addition, the present study demonstrated that FTO expression was significantly increased in the livers of non-alcoholic steatohepatitis (NASH) patients and in a rodent model of NASH (Lim et al., 2016). Genetic silencing of *Fto* had a protective effect on palmitate-induced oxidative stress, endoplasmic reticulum (ER) stress, and apoptosis *in vitro* (Lim et al., 2016). Inflammation is a characteristic of Metabolic syndrome (MetS). FTO may serve as a risk factor for MetS and inflammatory markers (Kraja et al., 2014). Thus, m6A methylation regulators can act as a link between inflammation and metabolic responses. These findings also warrant further investigation.

### Autoimmune Diseases and Infections

Inflammation is primarily caused by immune cells and cytokines. Inflammatory cells are composed of various immune cells, including neutrophils, macrophages, lymphocytes, and plasma cells. Cytokines are composed of inflammatory mediators, such as interleukin-1β (IL-1β), IL-6, and tumor necrosis factor-α (TNF-α) (Medzhitov, 2008). The presence of m6A modifications has been reported in both autoimmune diseases and infections.

Peripheral dendritic cells (DCs) mature in response to microbial products or inflammatory signals and then upregulate CC-chemokine receptor 7 (CCR7). m6A modification plays a critical role in DC-dependent inflammatory response. CCR7 stimulation upregulates lnc-Dpf3 by removing m6A modification to prevent RNA degradation (Liu et al., 2019). Lnc-Dpf3 feedback restrains CCR7-induced DC migration and inflammatory response. Macrophages play an important role in various chronic diseases. Recent studies have shown that m6A modification is involved in regulating macrophage phenotype. Silencing the m6A “reader” YTHDF2 increases MAP2K4 and MAP4K4 mRNA expression levels by stabilizing mRNA transcription, which in turn activates MAPK and NF-κB signaling pathways, further inducing the expression of pro-inflammatory cytokines and aggravating inflammatory response in LPS-stimulated RAW 264.7 cells (Yu et al., 2019). Rheumatoid arthritis (RA) is a chronic inflammatory autoimmune disease characterized by the progressive destruction of the articular cartilage and bone (Scott et al., 2010). Studies have shown that m6A modification is involved in RA pathogenesis. It has been reported that METTL3 expression significantly increased in RA patients and is positively correlated with CRP and ESR, the two common markers of RA disease activity (Wang J. et al., 2019). In addition, an *in vitro* study showed that LPS stimulation promoted the expression and biological activity of METTL3 in macrophages. However, overexpression of METTL3 can attenuate LPS-induced inflammation through the NF-κB signaling pathway (Wang J. et al., 2019).

Commensal bacteria, especially gut microbiota, play a role in critical physiological functions such as host metabolism, immune system development, and even behavior (Sommer and Backhed, 2016). Metabolites and fermentation products from gut microbiota have been reported to mediate intestinal effects in the host by regulating transcription and epigenetic modifications (Koh et al., 2016; Agus et al., 2018). Variations in the gut microbiota correlate with m6A modifications in the cecum. METTL16 is another N6-adenosine-methyltransferase (Pendleton et al., 2017; Shima et al., 2017; Warda et al., 2017). A previous study reported that METTL16 expression decreases in the absence of microbiota. The target mRNA, *Mat2a*, which encodes S-adenosylmethionine synthase, is less methylated (Jabs et al., 2020). FTO deletion in mice resulted in changes in bacterial characteristics associated with reduced inflammation, such as a higher abundance of *Lactobacillus* and a lower content of Porphyromonadaceae and *Helicobacter* (Sun et al., 2019). *Helicobacter pylori* interacts with gastric epithelial cells to induce gastric inflammation and epithelial damage. The expression of WTAP has been confirmed to be downregulated in AGS cells stimulated by *H. pylori* (Kim et al., 2007). Dental pulp inflammation is a common public health problem caused by oral bacterial infections. It has been reported that m6A levels and METTL3 expression are increased in LPS-stimulated human dental pulp cells (HDPCs). METTL3 deletion promotes the expression of MyD88S in HDPCs induced by LPS, reduces the expression of inflammatory cytokines, and inhibits the activation of the NF-κB and MAPK signaling pathways (Feng et al., 2018). These studies suggest that epitranscriptomic modifications act as an additional level of interaction between commensal bacteria and their host, affecting pathways related to inflammation and antimicrobial responses.

### Cancer

Inflammation is associated with the development of a cancer-prone microenvironment. Many types of tumors occur in these inflammatory microenvironments, and the inflammatory state can be pro-tumorigenic (Singh et al., 2017). Studies have shown that a disruption in the balance between pro-and anti-inflammatory mechanisms leads to chronic inflammation, which can induce tumor initiation and progression (Seruga et al., 2008). As inflammation progresses, it promotes the production of tumor-promoting cytokines (Singh et al., 2017) such as IL-11 (Bollrath et al., 2009), IL-1β (Tu et al., 2008), and IL-6 (Grivennikov et al., 2009; Singh et al., 2017), which support the survival, growth, and metastasis of tumor cells (Garg et al., 2012). Recently, numerous studies have suggested an association between m6A modifications and pro-inflammatory genes in the tumor microenvironment.

In the complex tumor microenvironment network, mesenchymal stromal cells (MSCs) play a key role in promoting tumor progression by interacting with tumor cells and other stromal cells (Pelizzo et al., 2018; Whiteside, 2018). The inflammation and autophagy-related gene P62 is highly expressed in most human tumor tissues (Moscat and Diaz-Meco, 2009; Ren et al., 2014). It has been demonstrated that P62 synergizes with TNF-α to promote the malignant transformation of human MSCs by forming insulin growth factor II (IGF-II) promoter-enhancer chromatin loops and increasing METTL3 occupancy on IGFII 3′ UTR, and upregulates the expression of H-Ras by harboring inflammation-related factors, such as TNFR1, CLYD, EGR1, NF-κB, TLR4, and PPARγ (Xin et al., 2018). In HCC, m6A modification levels and mRNA expression are increased. A recent study found that the deletion of the m6A “reader” YTHDF2 in human HCC cells or mouse hepatocytes aggravates inflammation, vascular reconstruction, and metastatic progression (Hou et al., 2019). YTHDF2 promotes the decay of m6A-containing IL-11 and serpin family E member 2 (SERPINE2) mRNAs, which are involved in inflammation-mediated malignant tumors and the destruction of normal blood vessels (Hou et al., 2019). Another study reported that LPS stimulation can increase m6A methylation of G-protein alpha-subunit (GNAS) mRNA to promote GNAS expression in HCC cells. Highly expressed GNAS promotes the growth and invasion of HCC cells by interacting with STAT3 (Ding et al., 2020). Intrahepatic cholangiocarcinoma (ICC) is the second most malignant type of primary liver cancer with a high degree of incidence and a very poor prognosis. FTO was found to regulate the inflammatory signaling pathways in clinical ICC samples. FTO knockdown inhibited the expression of NR5A2, which is a nuclear receptor related to pancreatic adenocarcinoma and transcriptionally regulates the expression of inflammatory genes (Rong et al., 2019). Moreover, the expression level of FTO was decreased in clinical ICC samples and inversely correlated with CA19-9 expression and micro-vessel density (MVD) (Rong et al., 2019). Thus, these results suggest that FTO may be a target for predicting the prognosis of ICC. It has recently been found that the evaluation of m6A modification patterns within individual tumors could predict tumor inflammation stage and prognosis in gastric cancer (Zhang et al., 2020). A low m6A score, characterized by increased mutation burden and activation of immunity, has a higher 5-year survival rate. However, activation of the stroma and lack of effective immune infiltration were observed in the high m6A score subtype and associated with poorer survival. Patients with a lower m6A score demonstrated significant therapeutic and clinical benefits. In addition, m6A modification of non-coding RNAs can affect tumor formation. METTL3 may have an oncogenic role in bladder cancer by positively modulating pri-miR221/222 maturation in an m6A-dependent manner (Han et al., 2019).

### Others

Many infectious diseases, such as osteomyelitis, osteoarthritis, and periodontitis, can disrupt bone homeostasis (Josse et al., 2015; Pacios et al., 2015; Maruotti et al., 2017). LPS is the main pathogenic factor in infectious bone destruction. Studies have revealed that METTL3 knockdown stabilizes Smad7 and Smurf1 mRNA transcripts via YTHDF2 to inhibit osteoblast differentiation and Smad-dependent signaling, and regulates MAPK signaling to activate inflammatory response to LPS (Zhang et al., 2019). A previous study emphasized the influence of genetic risk for obesity and osteoarthritis, but the association is only regulated by the effect on BMI, which is consistent with what is known about the biology of the FTO gene (Panoutsopoulou et al., 2014).

Neuronal fate after ischemic stroke is determined by intricate biochemical and molecular events, including oxidative stress, ER stress, apoptosis, and inflammation (Mehta et al., 2007). The brain has been shown to have higher m6A abundance than other mammalian organs (Meyer et al., 2012). Thus, post-transcriptional regulators fine-tune the post-ischemic pathophysiology. An *in silico* analysis demonstrated increased m6A methylation in major inflammatory pathways, including IL-6 cytokine, TNF, TLR, and NF-κB signaling pathways. The expression of major neuronally localized m6A demethylase FTO was downregulated, which presumably leads to decreased demethylation of m6A tagged RNAs in the post-stroke brain. However, ALKBH5 expression was unaltered in the ischemic brain (Chokkalla et al., 2019). In addition, a decrease in total m6A demethylase activity was reported in the study, indicating that FTO downregulation might be responsible for the decreased m6A demethylation in the post-stroke brain (Chokkalla et al., 2019). Therefore, it is plausible that ischemia-induced loss of FTO might be a modulator of post-ischemic secondary brain damage.

As a major cause of mortality worldwide, cardiovascular and metabolic diseases have a higher risk of occurrence in people with high levels of systemic inflammation and are independent of traditional cardiometabolic risk factors (Danesh et al., 2004). A recent study evaluated the association between inflammation and cardiometabolic phenotypes. The genetic overlap includes FTO, which has been confirmed to have an effect on CRP (Ligthart et al., 2015). Owing to its role in obesity-predisposing genetic variants, FTO has been shown to have nominally significant associations with cardiovascular disease and is referred to as a cardiovascular biomarker (He et al., 2010).

Polycystic ovary syndrome (PCOS) is a highly heterogeneous disease of the reproductive system that is associated with cellular metabolism and chronic inflammation (Ojeda-Ojeda et al., 2013). It has been reported that SNP rs9939609 of FTO is significantly related to PCOS (Zhao et al., 2016). Notably, FTO is a risk factor for PCOS that is independent of its correlation with BMI or obesity (Zhao et al., 2016). Therefore, the FTO gene has a profound effect on PCOS and is considered a candidate gene for this disorder.

### Therapeutic Potential

Recent studies have demonstrated that deregulation of m6A regulators is linked to cancer therapy resistance. Chemotherapeutic drugs modulate m6A regulators to stabilize the mRNA of oncogenes and induce chemoresistance (Shriwas et al., 2020). The tumor microenvironment activated by m6A regulators induces resistance to immunotherapy in cancer cells (Yang et al., 2019; Yi et al., 2020). Radiation stabilizes the mRNA of cancer stem cells by modulating the m6A regulator (Taketo et al., 2018; Visvanathan et al., 2018). As m6A plays an important role in therapy resistance, the targeted inhibition of m6A regulators may lead to better outcomes.

**FIGURE 4:**
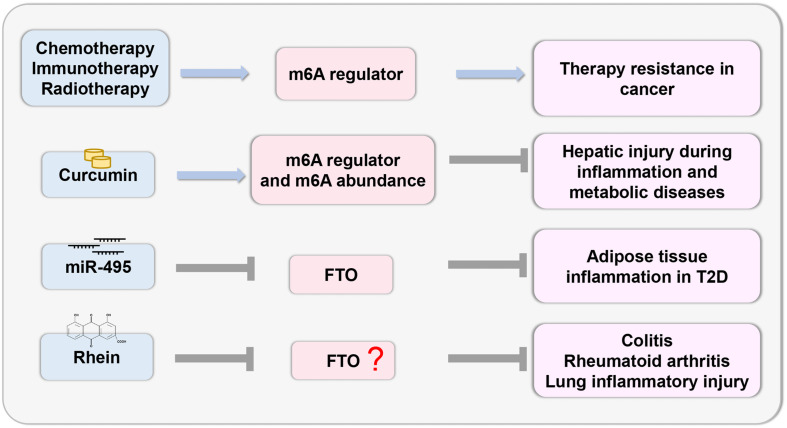
Therapeutic potential based on m6A. The study of epigenetic changes in inflammatory response provides the possibility to develop effective drugs with specific targets based on the m6A regulator.

The study of epigenetic changes in inflammatory response provides the possibility of developing effective drugs with specific targets (Figure 4). Recently, curcumin, a yellow polyphenolic pigment derived from the spice turmeric (*Curcuma longa*), has been reported to protect against LPS-induced liver injury and lipid metabolism disorders. Dietary curcumin can regulate the mRNA expression of METTL3, METTL14, ALKBH5, FTO, and YTHDF2 and increase m6A abundance, which has a protective effect on hepatic injury due to inflammation and metabolic diseases (Lu et al., 2018). In T2D, miR-495 inhibited FTO expression, thus promoting the transformation of macrophages into M1-type pro-inflammatory macrophages, and aggravated insulin resistance and adipose tissue inflammation (Hu et al., 2019a). Thus, this research provides a theoretical basis for targeted treatment. Notably, there are few inhibitors that specifically target m6A regulatory proteins. The natural product Rhein, which is identified as a demethylase FTO inhibitor, competitively binds to the FTO active site *in vitro* (Chen et al., 2012). Rhein has been found to suppress ATP-triggered inflammatory responses in fibroblast-like synoviocytes of rheumatoid rats and ameliorate experimental colitis (Hu et al., 2019b; Wu et al., 2020). In addition, Rhein treatment can inhibit inflammatory lung injury induced by the human respiratory syncytial virus (Shen et al., 2019). However, whether these protective effects are mediated by m6A modifications remain to be investigated. Thus, more efficacious medicines and novel therapeutic strategies related to m6A modification should be explored.

## Perspectives and Conclusion

Although m6A has become the focus of numerous studies, the mechanism by which m6A modifications affect inflammation in human diseases remains unclear. For instance, human HCC exhibits a characteristic increase in m6A modification, which is related to inflammation-mediated malignancy (Hou et al., 2019). Meanwhile, LPS stimulation can increase m6A levels and trigger inflammatory cytokine production and pathways in liver tumors and dental pup inflammation (Feng et al., 2018; Ding et al., 2020). Mechanistically, it has been reported that silencing *Mettl3* could maintain LCFA absorption by blocking the TRAF6-dependent inflammation response (Zong et al., 2019). However, METTL3 depletion enhances the expression of pro-inflammatory cytokines and promotes the activation of MAPK and NF-κB signaling pathways in osteoblasts (Zhang et al., 2019). In RA, LPS stimulation upregulates the expression and biological activity of METTL3 in macrophages, while METTL3 overexpression inhibited the inflammatory response (Wang J. et al., 2019). These data suggest that the associations between m6A modifications and inflammatory responses are highly dependent on the context of specific diseases and signaling molecules, especially as many m6A regulators are still being investigated to identify novel functions.

In summary, RNA methylation plays essential roles in various inflammatory disease states, including autoimmunity, infection, metabolic disease, cancer, neurodegenerative disease, heart disease, and bone disease via multiple mechanisms. Nevertheless, our understanding of this phenomenon is still incomplete. A major challenge is to study the precise functions of each m6A regulatory factor in different cells during the inflammatory response at various stages of disease development. In addition, other components of m6A modification remain to be discovered. Importantly, the development of scientific technology, such as the application of m6A mapping methods and m6A editing tools, will be immensely helpful for future research on m6A modification at the single nucleotide level.

## Author Contributions

TX and KS: concept and design. JL: drafting the manuscript. All authors: revising the manuscript content and approving the final version of the manuscript.

## Conflict of Interest

The authors declare that the research was conducted in the absence of any commercial or financial relationships that could be construed as a potential conflict of interest.
